# β-Catenin: A Metazoan Filter for Biological Noise?

**DOI:** 10.3389/fgene.2019.01004

**Published:** 2019-10-16

**Authors:** Saba Rezaei-Lotfi, Neil Hunter, Ramin M. Farahani

**Affiliations:** ^1^IDR/Westmead Institute for Medical Research, Sydney, NSW, Australia; ^2^Faculty of Medicine and Health, University of Sydney, Sydney, NSW, Australia

**Keywords:** β-catenin, biological noise, transcriptional regulation, cell cycle, translational regulation

## Abstract

Molecular noise refers to fluctuations of biological signals that facilitate phenotypic heterogeneity in a population. While endogenous mechanisms exist to limit genetic noise in biological systems, such restrictions are sometimes removed to propel phenotypic variability as an adaptive strategy. Herein, we review evidence for the potential role of β-catenin in restricting gene expression noise by transcriptional and post-transcriptional mechanisms. We discuss mechanisms that restrict intrinsic noise subsequent to nuclear mobilization of β-catenin. Nuclear β-catenin promotes initiation of transcription but buffers against the resultant noise by restraining transcription elongation. Acceleration of cell cycle, mediated *via* Wnt/β-catenin downstream signals, further diminishes intrinsic noise by curtailing the efficiency of protein synthesis. Extrinsic noise, on the other hand, is restricted by β-catenin–mediated regulation of major cellular stress pathways.

## Introduction

In biological systems, noise can be defined as variability in transmission of a signal between two sources. The variability often manifests in the unpredictability of emergence of a certain outcome. Such unpredictability is a direct consequence of variations in biological signals that maintain the genotype/phenotype continuum. Within physiological limits, phenotypic heterogeneity that is driven by biological noise enriches the population and provides a basis for natural selection. It must be emphasized, however, that phenotypic heterogeneity mediated by noise contrasts sharply, and acts synergistically, with encoded (genotypic) heterogeneity. While genetic noise could propel phenotypic diversity in the absence of genotypic differences, the existence of such genomic variability amplifies the impact of noise.

Non-encoded induction of heterogeneity by genetic noise facilitates adaptation to stressors. Induction of competence in *Bacillus subtilis* is a prime example of such noise-mediated adaptation to stressors (Spizizen, 1958). In the transient state of competence, *B. subtilis* internalizes exogenous DNA and integrates it into the genome. Suel et al. demonstrated that competence corresponds to an unstable state that is driven by stressor-mediated induction of gene expression noise while the competent cells eventually return to the non-competent state (Suel et al., 2006). Similar to prokaryotes, genetic noise is instrumental in development of metazoans. Spatial patterning of the retinal color-vision mosaic during development of *Drosophila melanogaster* is instructed by stochastic transcriptional bursts that invoke probabilistic resolution of dichotomous fate outcomes (Mikeladze-Dvali et al., 2005). It becomes evident that induction or suppression of genetic noise is strictly regulated. Notably, molecular mechanisms that regulate gene expression noise in metazoans remain largely unknown. Herein, we review the evidence for regulation of gene expression noise by a major cytoskeletal protein, β-catenin.

## Biological Definition of Noise

**Figure 1:**
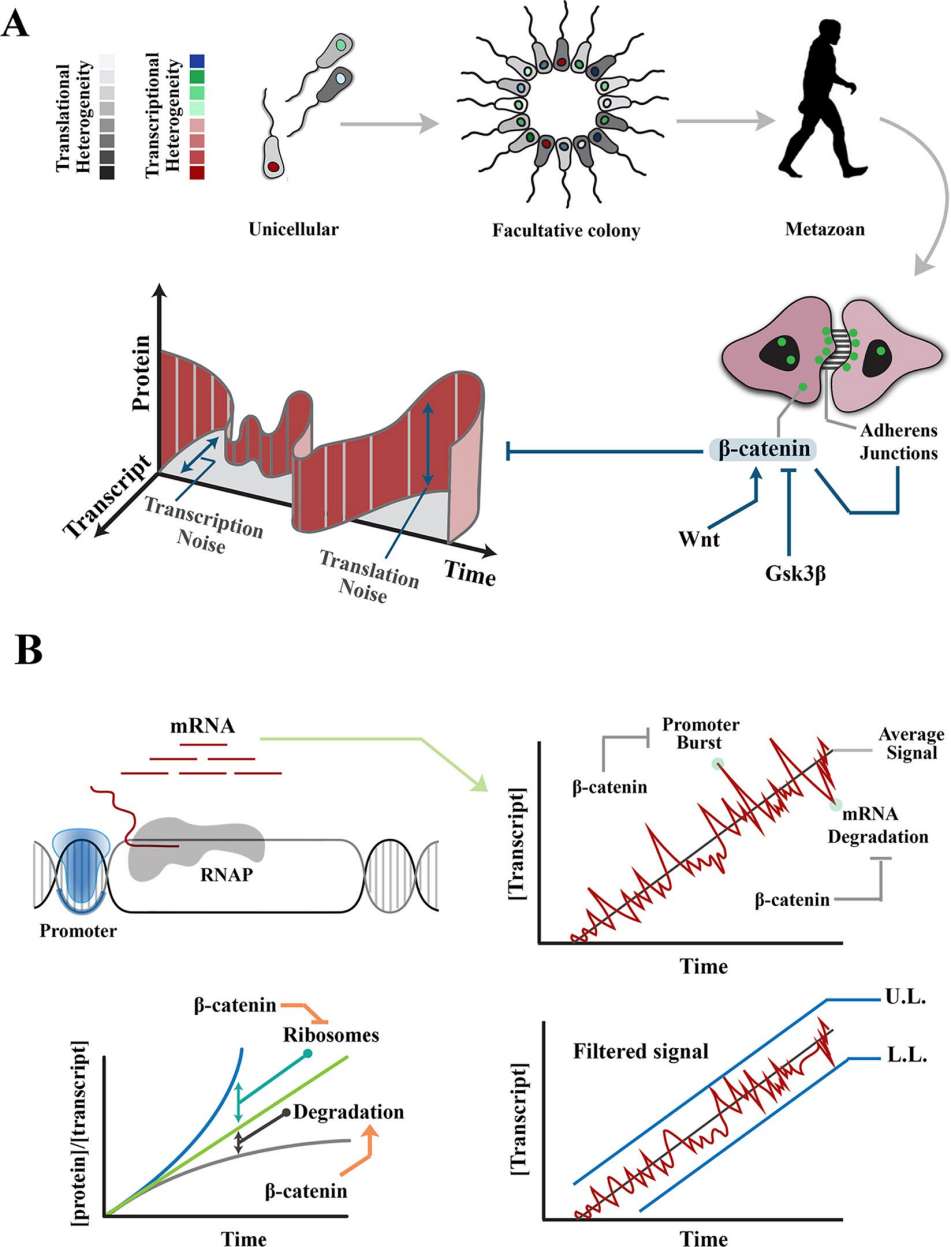
Regulation of intrinsic noise * via* β-catenin occurs at various levels during transmission of a signal. **(A)** Transition from unicellular life forms into multicellularity was predicated upon existence of mechanisms that regulate gene expression noise during developmental self-organization. The evolution of β-catenin not only facilitated cell–cell communication and multicellular self-organization but also provided a mechanism to control gene expression noise at transcription and translation levels. **(B)** Images demonstrate rising concentration of a transcript after activation of the associated promoter. Stochastic events, such as promoter-driven transcriptional burst or mRNA degradation, could potentially alter transcript availability, independent of upstream events that induce the signal, and lead to noise in transmitted signals. Likewise, increased efficiency of ribosomal translation and post-translational degradation of a protein (e.g., by autophagy) could alter the ratio of transcript to protein and introduce noise into the system. Interactions of β-catenin reduce transcription-related noise by buffering against promoter-driven transcriptional bursts and stabilizing mRNAs (right). As such, β-catenin stabilizes upper and lower limits of transcriptional noise. Further, β-catenin reduces translation-related noise by shortening G1 phase (and hence curtailing protein synthesis) and amplifying protein degradation (lower left). UL, upper limit; LL, lower limit.

Broadly, molecular noise refers to processes that introduce stochasticity into monostable deterministic outcomes in a cell or a population of cells (Samoilov et al., 2005; Samoilov et al., 2006). In this review, however, noise refers to cell–cell variability of gene expression, measured at protein level, as originally proposed by Elowitz et al. (2002). Therefore, noise in synthesis of a particular protein can be defined as the standard deviation divided by the average concentration of that protein (signal) in a population of cells. Gene expression noise can be classified into two broad categories based on the origin as extrinsic noise or intrinsic noise (Raser and O’Shea, 2005). Intrinsic noise results from fluctuations of biological signals that occur within an individual cell as the signal is generated or transmitted (**Figure 1A**). These fluctuations are cumulative consequences of the inherent stochasticity of transcription dynamics, post-transcriptional interactions and translational variability, (Julicher and Bruinsma, 1998) that alter the strength of a biological signal independent of external influences. Stochastic events, such as replication–transcription conflict (Garcia-Muse and Aguilera, 2016) and RNA polymerase backtracking mediated by R-loop formation (Skourti-Stathaki, 2014), are examples of events that invoke intrinsic noise in an individual cell. Broadly, extrinsic noise refers to cell–cell variability in gene expression that is communicated by fluctuations in other cellular components (e.g., RNA polymerase availability, mitochondrial activity, etc.) and affects multiple genes at the same time (Volfson et al., 2006). In the present context, extrinsic noise may be induced by exogenous events, e.g., environmental stressors (Mitosch et al., 2017). Herein, we discuss the activities of β-catenin in regulating extrinsic and intrinsic noise separately. Prior to such discussion, an overview of β-catenin activity and signaling is provided.

## β-Catenin Structure and Activity

Catenin-β is a cytoskeletal protein with dual functionality; apart from stabilization of the cytoskeleton, it is involved in the regulation of transcription (MacDonald et al., 2009). As a cytoskeletal protein, β-catenin binds to cadherin-based junctional complexes and stabilizes physical association of adherens junctions and the cytoskeleton (McCrea et al., 1991; Brembeck et al., 2006; Nelson, 2008). Unbound free cytoplasmic β-catenin is tightly regulated by a destruction complex that recruits the protein and degrades it subsequent to phosphorylation by Gsk-3β (Wu and Pan, 2010). Due to constant degradation, β-catenin has a short half-life of ≈1 h (Salic et al., 2000). Free cytoplasmic β-catenin can, however, escape phosphorylation and subsequent degradation. Upon stimulation by Wnt, disheveled interferes with activity of the destruction complex (Fiedler et al., 2011). Subsequently, rescued free β-catenin is shuttled into the nucleus where it binds to the *trans*-activation partner, TCF3/LEF1 (Behrens et al., 1996) to activate transcription from specific genomic loci (He et al., 1998). Nuclear localization of β-catenin and the resultant *trans*-activation are two parallel events that synergistically regulate intrinsic and extrinsic noises in gene expression.

## Modulation of Intrinsic Transcriptional Noise by β-Catenin

Intrinsic noise in gene expression results from probabilistic variation in transcriptional and translational outputs. Transcription-related noise manifests as stochastic amplification or attenuation of transcriptional output, independent of the stimulus that has triggered the initiation of transcription. Such variability can occur during initiation or elongation steps of transcription. At the initiation stage, promoter-driven fluctuations of RNA polymerase-II (RNAP-II) activity are a major source of intrinsic noise (Raser and O’Shea, 2005; Blake et al., 2006). The promoter fluctuations lead to bursts of transcription initiation and enhance the likelihood of cell–cell variability among a group of identical cells (Blake et al., 2006). However, inefficient transcription elongation could buffer such promoter-driven intrinsic noise by restricting the flow-on effect on downstream transcripts (Raser and O’Shea, 2005). It is by strategic positioning at the interface of transcription initiation and elongation that β-catenin can regulate intrinsic transcriptional noise (**Figure 1B**).

β-catenin reshapes the global transcriptional landscape by reducing processivity of RNAP-II (Daugherty et al., 2014). Inhibition of RNAP-II occurs by combined activities of α-catenin and β-catenin that does not affect promoter loading of the transcriptional initiation complex. Altered processivity of RNAP-II and the resultant inefficiency of transcription reduces transcriptional noise by diminishing the global availability of transcripts and buffering the transcribed mRNAs against promoter-driven fluctuations during transcription initiation (Ko, 1991; Raser and O’Shea, 2004; 2005). Combined activities of α-catenin and β-catenin can also attenuate global transcriptional activity by non-canonical induction of DNA double-stranded DNA breaks (Serebryannyy et al., 2017). The repair of DNA damage could delay transcription by ≈30 min in non-homologous end-joining and ≈7 h in homologous recombination (Mao et al., 2008). This is a significant delay as the rate of transcription is 10 to 100 nt/s, and thus, it takes ≈10 min for a mammalian gene of 10 kbp to be transcribed (Shamir et al., 2016). Negative regulation of transcription elongation by induction of DNA cleavage could powerfully reduce the global transcriptional profile, and hence, the intrinsic noise in gene expression that results from fluctuations of promoter activity. β-Catenin can also curtail transcriptional activity *via* binding to the chromatin remodeling factor, CHD8 (Thompson et al., 2008). By these activities β-catenin controls bursts of transcriptional output. However, degradation of transcripts (Houseley and Tollervey, 2009) could also introduce cell–cell variability in gene expression. Notably, β-catenin regulates the stability of mature transcripts (Ross, 1996). This particularly applies to transcripts, such as cyclooxygenase-2 (COX-2), that are constantly and rapidly degraded by default and are only stabilized in acute phase responses, such as inflammation. β-catenin interacts with the 3′-untranslated region of several key transcripts leading to stabilization of the mRNA and resultant restriction of transcriptional noise (Briata et al., 2003; Lee et al., 2007).

By these mechanisms, β-catenin reduces cell–cell variability and hence the standard deviation of molecular signals in a population. Reduction of the standard deviation can be interpreted as establishing the upper and lower limits of transcriptional noise. In other words, standard deviation (S) of a protein copy number (x_i_) in a population of cells would approach a minimum (S_min_) if the copy numbers in individual cells converge toward a central value:

$$S=\sqrt{\frac{1}{n-1}\sum_{i=1}^{n}{\left(x_{i}-\bar{x}\right)}^{2}}$$

$$\min(x_{i})\to \bar{x}\text{ \& }\max(x_{i})\to \bar{x}\;\Rightarrow \text{ S}\to {\text{S}}_{\min}$$

The lower limit (min *x*_i_) is established by stabilizing the transcribed mRNAs. The upper limit (max *x*_i_) is maintained by β-catenin–mediated buffering of transcriptional bursts as described above.

## Modulation of Translational Noise by β-Catenin

The transcription factor c-Myc (c-myelocytomatosis oncogene) is one of the key genes that is *trans*-activated by β-catenin signaling (He et al., 1998). Myc, in turn, stimulates transcription of genes involved in growth and proliferation (Zeller et al., 2006). It is, therefore, not surprising that ribosome biogenesis and global protein synthesis are controlled by transcriptional activity of Myc (van Riggelen et al., 2010). Myc binds to E-box sequences in the promoters of active rDNA clusters and directly regulates the RNAPI-mediated transcription of 18S, 5.8S, and 28S rRNAs (Grandori et al., 2005). Myc also orchestrates RNAPII-mediated transcription of ribosomal proteins (Boon et al., 2001). In addition, translation initiation factors, eIF4E, eIF2α, eIF4AI, and eIF4GI, are regulated by transcriptional activity of c-Myc (Schmidt, 2004). While efficient translation is known to invoke translational noise (Ozbudak et al., 2002), coupling of such efficient translation to G1 shortening (Farahani et al., 2019) restricts and reduces noise. This is because feedback from ribosome biogenesis is integrated into cell cycle and controls accelerated progression through G1 phase of cycle (Bernstein et al., 2007). β-catenin is the key molecule that couples ribosome biogenesis and cell cycle dynamics, as explained below.

**Figure 2:**
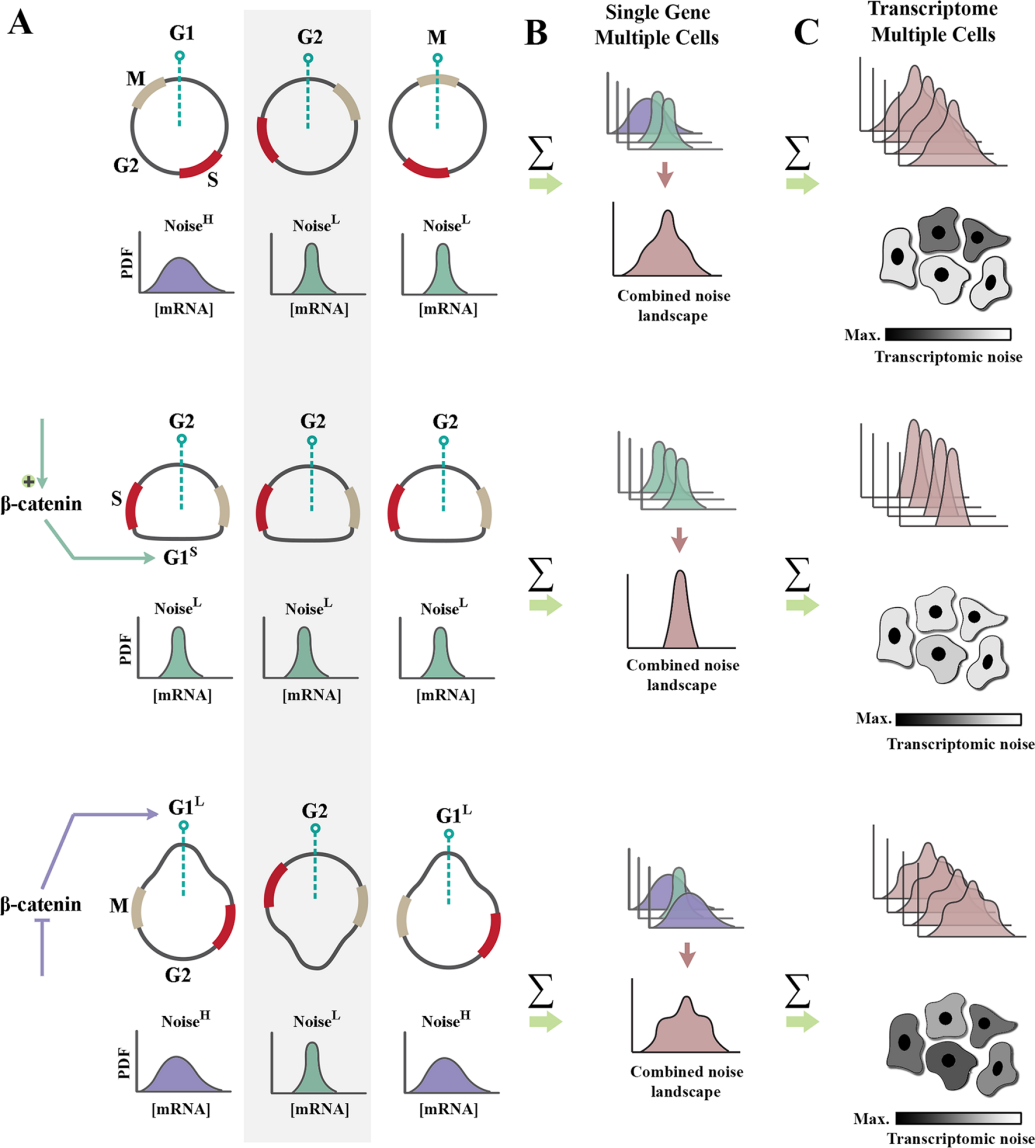
Regulation of cell cycle by β-catenin modulates genetic noise at population level. **(A)** Schematic graphs demonstrate the cell cycle state of n = 3 random cells at time t (dotted green line indicates time point t). Partially synchronized cells are at G1, G2, and M phases of cycle (top). Shortening of G1 phase (G1^S^) by amplification of β-catenin leads to complete synchronization of the population (middle). In contrast, lengthening of G1 phase (G1^L^) *via *inhibition of β-catenin triggers de-synchronization of the population (bottom). Note that shortening of noisy G1 decreases the intrinsic noise (defined as standard deviation/mean intensity of a signal in a defined temporal window). Cells with a high transcriptional noise (G1^L^ cells labeled as noise^high^) exhibit broad distribution of transcription rate as opposed to G1^S^ cells with a low transcriptional noise (noise^low^). Lengthening of G1 phase enhances the temporal stochasticity of a signal in individual cells. **(B)** The schematic graphs demonstrate theoretical distributions of transcription rate at a specific temporal window for the cells in left panel. Reduced temporal variability and cell cycle synchronization lead to the reduction of gene expression noise at a population level (combined noise landscape) that is associated with a single gene. **(C)** The schematic graphs demonstrate theoretical alignment of combined noise landscapes that belong to multiple transcripts in a putative transcriptome. Reduced transcriptomic noise leads to cellular homogeneity and robustness of developmental self-organization in metazoan animals.

Upon association of β-catenin with DNA-binding partners, repressive activity of TCF/LEF transcription factors is abolished, and transcription is initiated (Wu et al., 2012). Two major drivers of G1 phase of cell cycle, cyclin-D1 (Shtutman et al., 1999) and c-Myc (He et al., 1998), are among those genomic loci *trans*-activated by nuclear β-catenin. As such, enhanced β-catenin signaling accelerates interphase by shortening G1 phase of cycle (**Figures 2A, B**). A further corollary of such activity is that it improves synchronicity of a cycling population (Dokumcu et al., 2018; Farahani et al., 2019). Synchronized and accelerated cycling has two important consequences (**Figure 2**). Synchronicity of cycling reduces population-level variability and genetic noise that results from such variability (Pauklin and Vallier, 2013). Further, acceleration of cycle by shortening of G1 reduces the length of time that individual cells spend in the “noisy” G1 phase of cycle (**Figures 1B** and **2**).

Shortening of G1 phase can reduce translation-related noise by another parallel mechanism. A major source of translation-related noise is temporal oscillation of the key E3 ubiquitin ligase complexes that regulate cell cycle: anaphase-promoting complex (APC/C), and Skp, Cullin, F-box–containing complex (SCF complex) (Vodermaier, 2004). The APC/C complex is active during M/G1 phases, and the SCF complex regulates S/G2 phases of cycle in a mutually exclusive manner. While the range of substrates directed to degradation by the APC/C complex is restricted (Thornton and Toczyski, 2003), the SCF complex has a broader substrate recognition capacity. As such, it is expected that noise would be suppressed more efficiently by SCF-mediated post-translational degradation of substrates during S/G2 phases. Therefore, it can be concluded that a truncated G1 induced by enhanced β-catenin signaling could potentially reduce translation-related noise (Farahani et al., 2019) by two parallel mechanisms, global reduction of protein synthesis and amplified ubiquitin-mediated protein degradation. β-catenin can also directly enhance degradation of other proteins by acting as an adaptor protein.

While β-catenin is a major target of glycogen synthase kinase 3β (Gsk-3β) and the associated destruction complex, other substrates are also directed to proteosomal degradation by activity of this complex (Kim et al., 2009). These include the cytoskeletal proteins α-actin, tenascin, myosin regulatory light chain, ribosomal proteins, such as rpL30 and some kinases, e.g., serine-threonine kinase-35. The specific role of β-catenin in this process is best illustrated by post-translational depletion of Hippo cascade mediators. Two important mediators of Hippo signaling cascade, Yap and Taz (Meng et al., 2016), participate in formation of the β-catenin destruction complex (Azzolin et al., 2014). Therefore, in Wnt^off^ cells, β-catenin acts as an adaptor that directs post-translational degradation of Hippo cascade mediators and hence reduces noise in this signaling pathway. In addition to interaction with intrinsic sources of noise, the buffering capacity of β-catenin is bolstered by its particular mode of communication with extrinsic inducers of gene expression noise.

## Extrinsic Inducers of Noise Interface With β-Catenin

On route to degradation, β-catenin is enzymatically altered by proteins including Gsk-3β that also sense cellular stress. Therefore, activation of stress adaptation pathways in response to genetic noise (Spizizen, 1958) could potentially modulate availability of β-catenin, as discussed below. In mammalian cells, stressors are sensed by Gsk-3β (Maurer et al., 2014), mammalian target of Rapamycin (mTOR) (Aramburu et al., 2014), and NF-kb (Mercurio and Manning, 1999) pathways. As noted previously, Gsk-3β is a major component of the β-catenin destruction complex (Wu and Pan, 2010). Stressors such as hypoxia (Roh et al., 2005) activate Gsk-3β leading to a reduced level of free cytoplasmic β-catenin. In parallel, stress-mediated inhibition of mTOR activity (Xie et al., 2011) enhances autophagic flux (Jung et al., 2010) and leads to depletion of cytoplasmic β-catenin by autophagy (Kuhn et al., 2015). Signaling by NF-kb, a sensor for oxidative stress (Schreck et al., 1992), results in ubiquitination and subsequent degradation of β-catenin (Chang et al., 2013). Hence, in response to stressors that exceed a certain threshold, e.g., DNA damage, free cytoplasmic β-catenin is depleted (Sadot et al., 2001) and cell cycle is arrested in the noise-prone G1 phase (Agarwal et al., 1995).

## Transcriptional Rewiring Enhances Buffering of Extrinsic Noise by β-Catenin

Cascade-level associations with other signaling pathways remarkably amplify the capacity of β-catenin in buffering genetic noise (**Supplementary Figure 1**). Herein, a few examples are provided. Downstream to TGF-β signaling, Smad-3 rescues β-catenin from degradation and assists nuclear localization of this protein (Zhang et al., 2010). Once in the nucleus, Smad-3 partners with Tcf/Lef to activate downstream targets of the Wnt/β-catenin signaling cascade (Aloysius et al., 2018). However, Smad-3 is also required for the antagonistic Notch-1 signaling pathway (Blokzijl et al., 2003; Zavadil et al., 2004). As such, association of Smad-3 with activated β-catenin will diminish the available pool of Smad-3 that can interact with Notch-1 and will lead to a reduced Notch-1 signaling output. Given the inhibitory impact of Notch-1 on β-catenin (Kwon et al., 2011), attenuation of Notch-1 signaling further amplifies the noise-filtering capacity of β-catenin. Sonic hedgehog signaling is another major partner of β-catenin. Not only do both Gsk3β and CK1α phosphorylate Gli3 (downstream mediator of Sonic hedgehog) (Tempe et al., 2006) but also the phosphorylated protein is recognized and degraded by β-TrCP generating the truncated Gli3R (Wang and Li, 2006), a trajectory that closely replicates that of β-catenin degradation. Further, released Gli3R inhibits β-catenin transcriptional activity by direct interaction with the C-terminal domain (Ulloa et al., 2007). Upon activation of β-catenin by inhibition of the associated destruction complex, the negative impact of GliR will also be abolished. In turn, this amplifies the noise-buffering activity of β-catenin. Activation of Hedgehog signaling can also suppress p53, a major inhibitor of β-catenin (Sadot et al., 2001), by activating Mdm2 (Abe et al., 2008). It may be concluded that network-level wiring of β-catenin to other signaling cascades moderates its activity and provides an excess capacity that could be unleashed to repress gene expression noise with higher efficiency.

## WNT as an Amplifier of Noise Buffering Capacity

As mentioned previously, binding of Wnt ligands to the associated receptor (Wnt^on^) blocks the destruction of β-catenin and enhances noise buffering capacity of the protein under special circumstances (Arias and Hayward, 2006). During ontogeny, this amplification of noise dampening becomes critical in temporal windows where genetic noise is not tolerated. The segmentation clock, for example, is tightly controlled by combined activities of Wnt and Notch signaling cascades during somitogenesis (Blokzijl et al., 2003; Zavadil et al., 2004). (Pourquie, 2003). As such, gene expression noise in the temporal domain could obscure the sharp boundary of segments due to interference in temporal oscillations of developmental cascades. In agreement with this notion, fuzzy somite boundaries are a major consequence of impaired Wnt signaling (Aulehla et al., 2003).

## Medical Implications of Altered Noise Buffering

Altered regulation of gene expression noise is an efficient pro-survival strategy utilized by neoplastic cells to resist applied therapies (Brock et al., 2015). Amplified noise enhances phenotypic heterogeneity and survival capacity of neoplastic cells in parallel to, but independent of, genotypic divergence (Spencer et al., 2009). Noise also improves phenotypic plasticity of neoplastic cells and their transformation into more resistant subtypes (Brock et al., 2015). MicroRNA-mediated amplification of signaling by β-catenin efficiently overrides and represses noise and cell–cell variability in cancer cells (Dokumcu et al., 2018). It is notable that the latter noise buffering activity of β-catenin overrides the cellular heterogeneity that arises from mutational heterogeneity of neoplastic cells (Dokumcu et al., 2018). As such, exogenous regulation of gene expression noise may provide a therapeutic opportunity to reprogram the sensitivity (Spencer et al., 2009) of neoplastic cells and reduce recurrence rates following therapy (Dokumcu et al., 2018).

## Evolutionary Remarks

From an evolutionary perspective, the activity of Wnt/β-Catenin in buffering genetic noise is compatible with the ancestral function of this cascade. Wnt ligands interact with Frizzled receptors to activate the downstream signaling cascade. Frizzled receptors share the basic structural organization of G protein-coupled receptors (GPCRs) (Malbon, 2004). In yeast, GPCRs control the mating pathway that is used for sexual reproduction (Dohlman and Thorner, 2001). During sexual reproduction, secreted pheromones interact with GPCRs and activate expression of pheromone-responsive genes, leading to growth arrest and synchronization of the entire population into the opposite mating type (Bardwell, 2004). In addition to population-level synchronization, repression of growth-related genes minimizes genetic noise at an individual cell level (Bardwell, 2004). Interestingly, there is significant overlap between components of the yeast pheromone response pathway and the metazoan Wnt cascade (Bardwell, 2004). Further, Frizzled receptors can replace yeast GPCRs and stimulate the yeast mating response pathway in the absence of added Wnt ligands thereby leading to synchronization of the entire population (Dirnberger and Seuwen, 2007).

## Concluding Remarks

It may be concluded that evolution of β-catenin, in addition to its documented role in organization of cytoskeleton, provided metazoan life forms with a capacity to modulate gene expression noise. Buffering of noise is required to stabilize the evolutionary adaptations that characterize various species. This feature, referred to as Waddington’s canalization (Siegal and Bergman, 2002), improves developmental robustness and hence the chance of survival of a species. While it remains to be proven, it is tempting to postulate that β-catenin may play a central role in canalization of developmental traits. This proposition is aligned to the recent findings demonstrating that β-catenin regulates self-organization of neural progenitor cells (Rezaei-Lotfi et al., 2019). Buffering of gene expression noise *via* β-Catenin could enhance robustness of developmental self-organization.

## Author Contributions

RF: conception of the work. SR-L and RF: extensive literature search and manuscript drafting. NH and RF: critical revision of the work. RF and NH: final version approval.

## Funding

This study was supported by NIDCR Grant R01 DE015272 and Australian National Health and Medical Research Council Grant 512524.3.

## Conflict of Interest

The authors declare that the research was conducted in the absence of any commercial or financial relationships that could be construed as a potential conflict of interest.
