# Supplementary material for: β-Catenin: A Metazoan Filter for Biological Noise?
Source: Front Genet. 2019 Oct 16;10:1004. doi: 10.3389/fgene.2019.01004 (PMC6805772; doi:10.3389/fgene.2019.01004)
Supplement: Supplementary file 1 [file DataSheet_1.pdf]

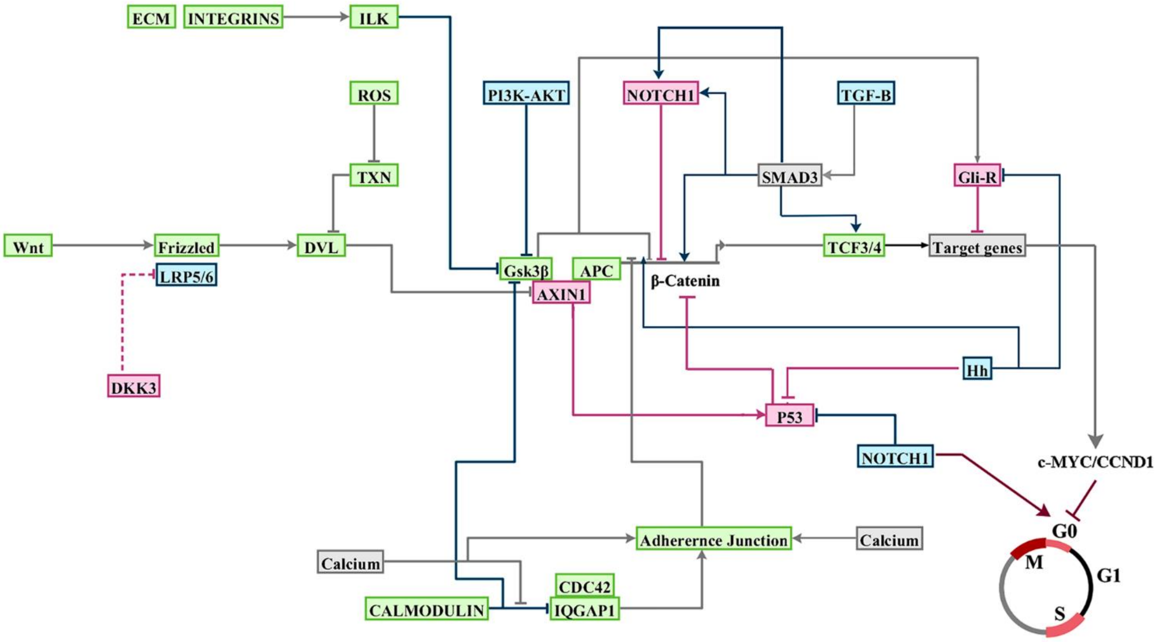

Supplementary Figure 1. Strategic wiring of β-catenin to other signaling cascades that boosts noise buffering capacity of the protein. Signaling by majority of pathways, e.g. sonic hedgehog (Hh), TGF-β, PI3K-Akt, amplify the transcriptional activity of β-catenin (blue lines). On the contrary, stress-related pathways, e.g. notch-1, p53, and gsk3β, reduce the availability of free-cytoplasmic β-catenin (red lines). Wnt signaling is the main amplifier of noise buffering capacity of β-catenin (green lines).
